# Prognostic impact of implantable cardioverter defibrillators and associated adverse events in patients with continuous flow left ventricular assist devices

**DOI:** 10.3389/fcvm.2023.1158248

**Published:** 2023-06-02

**Authors:** Jonas Pausch, Julian Mersmann, Oliver D. Bhadra, Markus J. Barten, Tobias Tönnis, Yalin Yildirim, Simon Pecha, Hermann Reichenspurner, Alexander M. Bernhardt

**Affiliations:** ^1^ Department of Cardiovascular Surgery, University Heart & Vascular Center Hamburg, University Medical Center Hamburg-Eppendorf, Hamburg, Germany; ^²^ Department of Cardiology, University Heart & Vascular Center Hamburg, University Medical Center Hamburg-Eppendorf, Hamburg, Germany

**Keywords:** implantable cardioverter defibrillator, ICD, left ventricular assist device, LVAD, Heart Failure, Ventricular arrhythmias

## Abstract

**Objectives:**

Implantation of implantable cardioverter defibrillators (ICD) reduces the risk of all-cause mortality in symptomatic heart failure (HF) patients with severe left ventricular (LV) dysfunction. Nevertheless, the prognostic impact of ICD therapy in continuous flow left ventricular assist device (LVAD) recipients remains controversial.

**Methods:**

162 consecutive HF patients, who underwent LVAD implantation at our institution between 2010 and 2019, were categorized according to the presence (***n* = 94, ICD-group**) or absence (***n* = 68, Control-group)** of ICDs. Apart from clinical baseline and follow-up parameters, adverse events (AEs) related to ICD therapy and overall survival rates were retrospectively analyzed.

**Results:**

Out of 162 consecutive LVAD recipients 79 patients (48.8%) were preoperatively categorized as Interagency Registry for Mechanically Assisted Circulatory Support (INTERMACS) profile ≤2. The prevalence of severe HF symptoms and preoperative use of short-term circulatory support devices (54.4% vs. 13.8%, *p* < 0.001) was higher within the Control-group, although baseline severity of LV and RV dysfunction was similar. Apart from an increased prevalence of perioperative right heart failure (RHF) within the Control-group (45.6% vs. 17.0%; *p* < 0.001), procedural characteristics and perioperative outcome were similar. Overall-survival during a median follow-up of 14 (3.0–36.5) months was similar within both groups (*p* = 0.46). During the first 2 years after LVAD implantation 53 ICD-related AEs occurred within the ICD-group. Thereof, lead-dysfunction occurred in 19 patients and unplanned ICD-reintervention in 11 patients. Furthermore, in 18 patients appropriate shocks without loss of consciousness occurred, whereas inappropriate shocks occurred in 5 patients.

**Conclusion:**

ICD therapy in LVAD recipients was not associated with a survival benefit or reduced morbidity after LVAD implantation. Conservative ICD-programming seems to be justified to avoid ICD-related complications and „awake shocks” after LVAD implantation.

## Introduction

Despite optimized medical therapy (OMT), the prevalence of ventricular arrhythmias (VAs) remains high in symptomatic HF patients with severe LV dysfunction (1). Particularly ischemic heart disease carries an increased risk of sudden cardiac death (SCD) (2), although myocardial fibroses due to non-ischemic dilated cardiomyopathy represents a potential substrate for life-threatening VAs as well (2). According to current HF guidelines, prophylactic ICD implantation to prevent SCD, is recommended in symptomatic HF patients with severe LV dysfunction, thereby reducing all-cause mortality (3, 4). Furthermore, secondary prevention ICD implantation in patients with documented sustained ventricular tachycardia (sVT) or survived SCD is associated with a survival benefit (5). Of note, ICD implantation in patients with end-stage HF awaiting heart transplantation (HTx) appears to result in an immediate and sustained reduction of mortality (6).

In addition to HTx, implantation of a durable continuous flow (cf) LVAD emerged to be a promising therapeutic option for symptomatic end-stage HF patients, to improve symptoms and reduce rehospitalization and premature death (7, 8). Nevertheless, despite LV support after LVAD implantation, the risk of VAs remains high, in particular in LVAD recipients with documented VAs prior to LVAD implantation (9). Although VAs are commonly well tolerated due to the ongoing cardiac output via cf LVAD, some LVAD recipients might develop right heart failure (RHF) (10). Therefore, particularly sVTs and ventricular fibrillation (VF) might result in an increased mortality risk in LVAD recipients, nevertheless conflicting data has been published (10, 11). Consequentially, in contrast to HF patients without LVAD support, the prognostic role of ICD therapy in cf LVAD recipients remains controversial (1).

## Patients and methods

### Ethical statement

The study confirms with the ethical guidelines of the Declaration of Helsinki. Due to the retrospective study design and anonymous data collection, written patients informed consent was waived as reflected and approved by our local ethical committee.

### Patients

**Figure 1:**
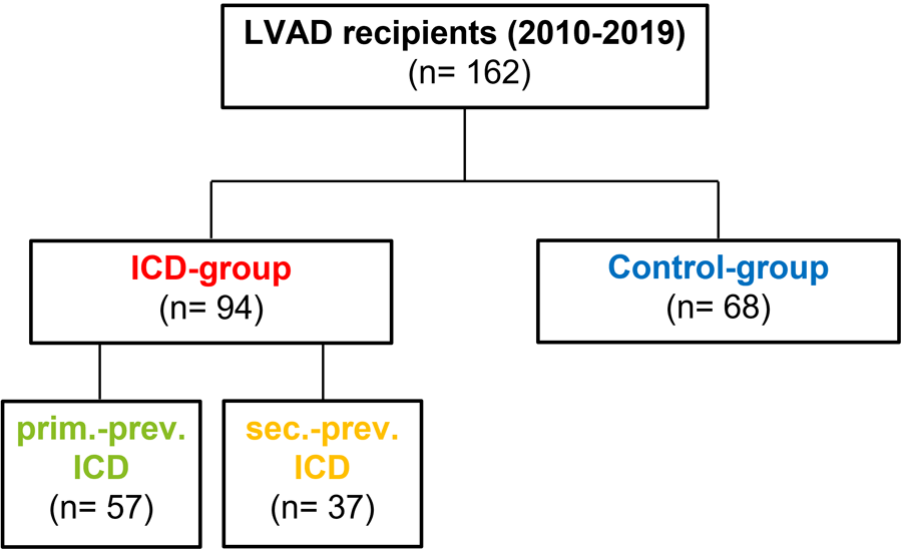
Study design—Categorization of LVAD recipients according to ICD presence or absence. 162 consecutive LVAD recipients were retrospectively categorized according to the presence (ICD-group; *n* = 94) or absence (Control-group; *n* = 68) of ICD prior to LVAD implantation. Subgroup analysis according to ICD-indication was performed and patients were therefore categorized in primary vs. secondary prevention ICD patients.

We retrospectively analyzed baseline, perioperative and follow-up (FU) data of 162 consecutive patients who underwent cf LVAD implantation at our institution between 2010 and 2019. According to current HF guidelines patients were selected for LVAD therapy as bridge to recovery, bridge to HTx or destination therapy. Patients undergoing concomitant durable right ventricular assist device (RVAD) implantation were excluded from the current analysis. Patients were categorized according to the presence (***n* = 94, ICD-group**) or absence (***n* = 68, Control-group)** of ICDs prior to LVAD implantation (Figure 1). Patients, who received ICD implantation after LVAD implantation were not included within the current analysis. AEs related to ICD therapy during the first two years after LVAD implantation and overall survival rates were retrospectively analyzed. Thereby, AEs were categorized in 4 subgroups, namely lead-dysfunction, ICD-reintervention, adequate and inadequate shocks. Lead-dysfunction was defined as any significant verifiable alteration in lead function after LVAD implantation and during follow-up, resulting in either significant reprogramming of ICD therapy (e.g., deactivation of LV stimulation in case of CRT-D or ICD-therapy) or surgical lead revision. According to interdisciplinary heart team discussion, individualized decision making determined the appropriate therapeutic option. ICD-reintervention was defined as any unplanned operative ICD-reintervention. Thereby, elective generator exchanges were excluded and not defined as AE. In contrast, re-interventions due to bleeding or pocket hematoma after elective generator replacement were defined as AE.

### Surgical setup and technique of LVAD implantation

LVAD implantation was performed under general anesthesia in a standard operating room by a dedicated surgical HF team, either via full-sternotomy, or minimally invasively using a partial upper sternotomy and simultaneous left-sided thoracotomy. Normothermic cardiopulmonary bypass (CPB) was used in all patients. Concomitant procedures (e.g., aortic valve replacement, tricuspid or mitral valve repair) were performed following our institutional standards without major modification during the study period. Implantation of a temporary RVAD (tRVAD) was done due to unsuccessful weaning from CPB or echocardiographic signs of RHF despite increased pharmacological inotropic support. Of note, no surgical procedure regarding ICD therapy (e.g., generator or lead replacement, lead removal) was performed during LVAD implantation.

### Statistical analysis

Baseline, perioperative and FU variables were retrospectively collected in a dedicated institutional LVAD database. Normally distributed continuous variables are presented as mean values and standard deviation. Median and interquartile ranges are used for non-normally distributed continuous variables. Absolute numbers and percentages are used for categorical variables. Unpaired t-test was used for between-group comparison of normally distributed numeric variables. Otherwise, Mann-Whitney U-test was used. Chi-squared or Fisher's exact test was used for between-group comparison of categorial variables according to the minimum expected cell size. Kaplan-Meier method was used for survival analysis. Timepoint 0 of the survival analysis reflects the date of LVAD implantation. Patients who underwent heart transplantation or LVAD explantation during the study period were censored. Patients at risk and cumulative deaths at each timepoint are displayed within the Kaplan-Meier plots. Univariable comparisons were performed by log-rank test. Multivariable adjusted Cox regression model was performed to exclude independent influence of all significantly (*p* < 0.01) differing baseline characteristics (i.e., ICMP, diabetes mellitus, prev. hemodialysis, serum-creatinin, serum GOT and serum GPT levels, baseline LVEDD, NHYA-class IV, prev. ECMO/Impella and INTERMACS ≤ 2). Results were considered statistically significant if p value was <0.05. IBM Corp. Released 2019. IBM SPSS Statistics for Windows, Version 26.0. Armonk, NY: IBM Corp. was used for all statistical analyzes. The data underlying this article will be shared on reasonable request to the corresponding author.

## Results

### Study population

A total of 162 consecutive symptomatic end-stage HF patients underwent cf LVAD implantation at our institution between 2010 and 2019. Patients were categorized according to the presence (*n* = 94, ICD-group) or absence (*n* = 68, Control-group) of ICDs prior to LVAD implantation (Figure 1).

Median age at the time of surgery was 57 (49-64) years in the whole cohort and 87.1% (141/162) of patients were male [Table 1]. Within the ICD-group 31.9% (30/94) of patients underwent single-chamber, 24.5% (23/94) dual-chamber, and 43.6% (41/94) biventricular ICD-implantation (i.e., cardiac resynchronization therapy-defibrillator CRT-D) prior to LVAD-implantation. No significant differences were present in ICD- vs. Control-group regarding most outcome relevant comorbidities, nevertheless the prevalence of ischemic cardiomyopathy was higher within the Control-group. Systolic LV and RV function at baseline was similar in both groups, although LV end-diastolic diameter (LVEDD) was significantly increased in the ICD-group (73 vs. 66 mm; *p* = 0.021). All patients were severely symptomatic and showed comparably elevated serum levels of natriuretic peptide (NT-pro-BNP) (i.e., 7375 (3553-13042) pg/ml in the ICD-group vs. 10994 (4714-19516) pg/ml in the Control-group; *p* = 0.12), although the rate of patients categorized as New York Heart Association (NYHA) class IV was higher in the Control-group. Furthermore, the prevalence of previous short-term circulatory support devices prior to LVAD implantation was higher within the Control-group 54.4% (37/68) vs. the ICD-group 13.8% (13/94) (*p* < 0.001) and Control-group patients were more frequently categorized as INTERMACS profile I or II [Table 1].

**Table 1:** Preoperative patient characteristics.

Variables	ICD-group	Control-group	*p*-Value
	(*n* = 94)	(*n* = 68)	
Age (years), median (IQR)	57 (49–64)	56 (47–65)	0.79
Male, *n* (%)	79 (84.0)	62 (91.2)	0.18
BMI, kg/m^2^, median (IQR)	27 (23–30)	26 (24–30)	0.35
Ischemic Cardiomyopathy, *n* (%)	36 (38.3)	41 (60.3)	0.006
Arterial hypertension, *n* (%)	39 (41.5)	24 (35.3)	0.47
Diabetes mellitus, *n* (%)	33 (35.1)	13 (19.1)	0.03
COPD > GOLD II, *n* (%)	14 (14.9)	5 (7.4)	0.15
Atrial fibrillation, *n* (%)	50 (53.2)	27 (39.7)	0.11
Previous stroke, *n* (%)	15 (16.0)	5 (7.4)	0.10
Previous hemodialysis, *n* (%)	8 (8.5)	16 (23.5)	0.008
Serum Creatinin level (mg/dl), median (IQR)	1.8 (1.3–2.3)	1.4 (1.1–1.9)	0.007
Serum NT-proBNP level (pg/L), median (IQR)	7375 (3553–13042)	10994 (4714–19516)	0.12
Serum GOT level (U/L), median (IQR)	29 (18–52)	68 (36–246)	<0.001
Serum GPT level (U/L), median (IQR)	27 (13–53)	77 (29–184)	<0.001
LVEF (%), mean ± SD	20.5 ± 6.3	19.9 ± 5.4	0.68
LVEDD (mm), mean ± SD	72.9 ± 10.9	66.0 ± 11.8	0.021
TAPSE (mm), mean ± SD	15.0 ± 4.5	15.2 ± 5.0	0.82
Destination therapy, *n* (%)	32 (34.0)	27 (39.7)	0.48
NYHA class IV, *n* (%)	56 (59.6)	56 (82.4)	0.002
Previous ECMO/Impella, *n* (%)	13 (13.8)	37 (54.4)	<0.001
INTERMACS class ≤ 2, *n* (%)	31 (33.0)	48 (70.6)	<0.001
Previous sternotomy, *n* (%)	28 (29.8)	14 (20.6)	0.19

BMI, body mass index; COPD, chronic obstructive pulmonary disease; ECMO, extracorporeal membrane oxygenation; GOLD, Global Initiative for Chronic Obstructive Lung Disease; INTERMACS, Interagency Registry for Mechanically Assisted Circulatory Support; LVEDD, left ventricular end-diastolic diameter; LVEF, left ventricular ejection fraction; NYHA, New York Heart Association; NT-pro-BNP, N-terminal pro-B natriuretic peptide; GOT, glutamic oxaloacetic transaminase; GPT, glutamic pyruvic transaminase; TAPSE, tricuspid annular plane systolic excursion.

### Procedural outcome

Patients were mainly treated via full-sternotomy (77.8%) using the Medtronic HVAD device (87.0%) [Table 2]. Normothermic CPB was used in all patients within both groups. The rate of concomitant procedures was higher within the Control-group [Table 2], mainly due to an increased prevalence of simultaneous ECMO and Impella explanation (54.4% (37/68) vs. 13.8% (13/94) (*p* < 0.001). Of note, 79 patients (48.8%) were preoperatively categorized as INTERMACS profile ≤2 (70.6% of Control-group patients vs. 33.0% of ICD-group patients; *p* < 0.001). There was no intraprocedural mortality within both groups. Postoperative ventilation time was similar in both groups, nevertheless perioperative RHF occurred more frequently within the Control-group (45.6% vs. 17.0%; *p* < 0.001) [Table 2]. 30-day mortality was 20.6% vs. 12.8% in the Control- vs. the ICD-group (*p* = 0.18).

**Table 2:** Periprocedural outcome.

Variables	ICD-group	Control-group	*p*-Value
	(*n* = 94)	(*n* = 68)	
Implantation of HVAD device, *n* (%)	81 (86.2)	60 (88.2)	0.43
Perioperative RHF, *n* (%)	16 (17.0)	31 (45.6)	<0.001
Concomitant procedures, *n* (%)	50 (53.2)	49 (72.1)	0.015
Duration of surgery (min), median (IQR)	290 (240–360)	303 (250–378)	0.25
Cardiopulmonary bypass time (min), median (IQR)	135 (110–170)	169 (108–207)	0.067
Postoperative ventilation time (h), median (IQR)	9 (5–12)	12 (5–31)	0.38
30-day mortality, *n* (%)	12 (12.8)	14 (20.6)	0.18

tRVAD, temporary right ventricular assist device.

### Overall-survival during FU

**Figure 2:**
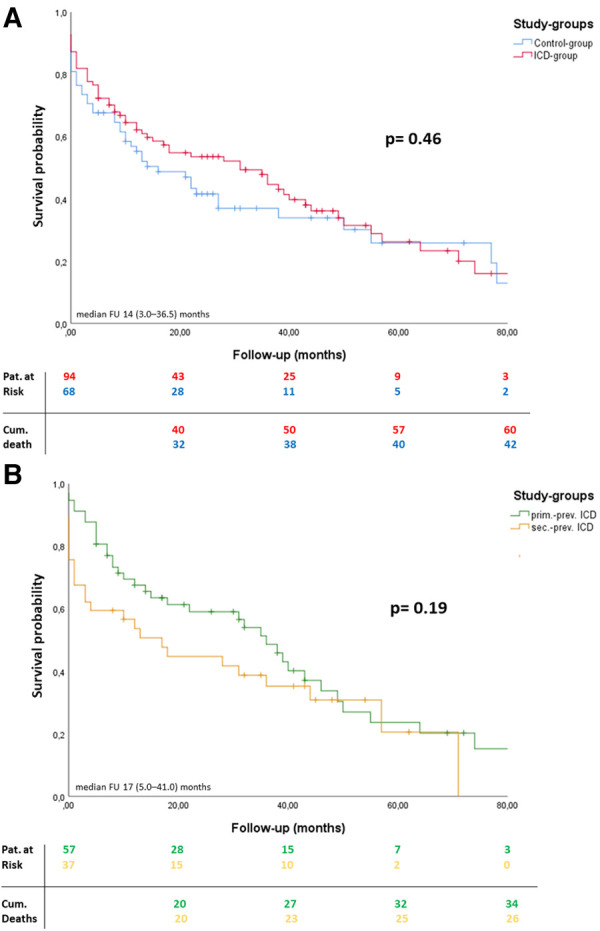
Overall survival during FU. Kaplan-Meier curves including patients at risk and cumulative deaths: Overall survival analysis during follow-up after LVAD implantation (timepoint 0) (**A, B**). *P*-values reflecting log-rank test between both groups. Median follow-up and interquartile range are reported in months.

During FU 20 patients (21.3%) within the ICD-group and 11 patients (16.2%) within the Control-group underwent HTx (*p* = 0.42). Furthermore, LV recovery and consecutive pump explantation was achieved in 10 patients (14.7%) within the Control-group. During a median follow-up of 14 (3.0–36.5) months overall survival was comparable between both groups (*p* = 0.46). Follow-up rate was 100%. (Figure 2A).

### ICD-related patient characteristics and adverse events during FU

Out of 94 ICD-group patients, 57 patients underwent primary prevention ICD implantation (primary prev. ICD-group), whereas 37 patients underwent secondary prevention ICD implantation (secondary prev. ICD-group) prior to LVAD implantation. Apart from a lower median age within the primary prev. ICD-group, there were no significant differences regarding patient demographics or outcome relevant comorbidities [Supplementary Table S1]. Furthermore, the extend of biventricular dysfunction, HF symptoms, and signs of secondary end-organ failure were similar in both subgroups [Supplementary Table S1]. Of note, approximately one third of patients within both subgroups were categorized as INTERMACS class ≤2, whereas the rate of concomitant tRVAD implantation was comparably favorable within both groups (*p* = 0.87). Median time to LVAD implantation after ICD implantation was similar in both subgroups (18 (8–44) months in the primary prev. ICD group vs. 18 (6–34) months in the secondary prev. ICD group; *p* = 0.2) (Table 3).

**Table 3:** Subgroup analysis—ICD related characteristics and adverse events during FU.

Variables	Primary prev. ICD	Secondary prev. ICD	*p*-Value
	(*n* = 57)	(*n* = 37)	
Time to LVAD implantation (months), median (IQR))	18 (8–44)	16 (6–34)	0.20
Patients with ICD related adverse events (ICD-AE), *n* (%)	25 (43.9)	15 (40.5)	0.75
Time to ICD-AE (months), median (IQR)	4.0 (1–15)	4.5 (1–12)	0.60
Lead dysfunction, *n* (%)	10 (17.5)	9 (24.3)	0.42
ICD reintervention, *n* (%)	8 (14.0)	3 (8.1)	0.38
Inappropriate shocks, *n* (%)	3 (5.3)	2 (5.4)	0.98
Appropriate shocks, *n* (%)	12 (21.1)	6 (16.2)	0.56
Ventricular arrythmias (VAs), *n* (%)	18 (31.6)	8 (21.6)	0.29
Time to VAs (months), median (IQR)	7 (4–15)	10 (6–17)	0.55

AE, adverse event; ICD, implantable cardioverter defibrillator; LVAD, left ventricular assist device; Vas, ventricular arrhythmias.

During the first 2 years after LVAD implantation 53 ICD-related AEs occurred in 40 ICD-group patients (42.5%). Median time to ICD-related AE was similar in both subgroups (4 (1–15) months in the primary prev. ICD group vs. 4.5 (1–12) months in the secondary prev. ICD group; *p* = 0.6). Lead-dysfunction occurred in 19 patients (10 (17.5%) patients in primary prev. ICD group vs. 9 (24.3%) patients in the secondary prev. ICD group; *p* = 0.42) and unplanned ICD-reintervention in 11 patients. Thereof, 6 patients required RV-lead revision due to immediate postoperative RV-lead dysfunction after LVAD-implantation. 2 patients underwent RV-lead revision due to lead dysfunction 20 and 21 months after LVAD-implantation. In addition, 2 patients had to undergo unplanned reoperation due to pocket hematoma after elective generator replacement due to battery depletion, whereas 1 patient underwent ICD-explantation due to pocket infection one month after elective generator replacement. Of note, within the first two years after LVAD-implantation additional 6 patients underwent elective generator exchange without complications.

In 18 patients, appropriate shocks without the loss of consciousness occurred, whereas inappropriate shocks occurred in 5 patients. Of note, deactivation of ICD-therapies (e.g., shock therapy) was performed in 9 patients according to patients' requests after experiencing several ICD shocks without the loss of consciousness. In one patient, ICD therapy was deactivated due to battery depletion. According to the decision of our multidisciplinary heart failure team, elective generator exchange was declined in this individual case to avoid the risk of potential bleeding complications and infection. At a median time of 9 (4–16) months after LVAD implantation relevant ventricular arrythmias (VAs) occurred in 26 (27.7%) patients (18 (31.6%) patients in primary prev. ICD group vs. 8 (21.6) patients in the secondary prev. ICD group; *p* = 0.38), whereof in 8 patients (30,8%) late VAs were ATP amendable. Overall survival during a median follow-up of 17 (5.0–41.0) months was similar in both subgroups (*p* = 0.19) (Figure 2B).

## Discussion

**Figure 3:**
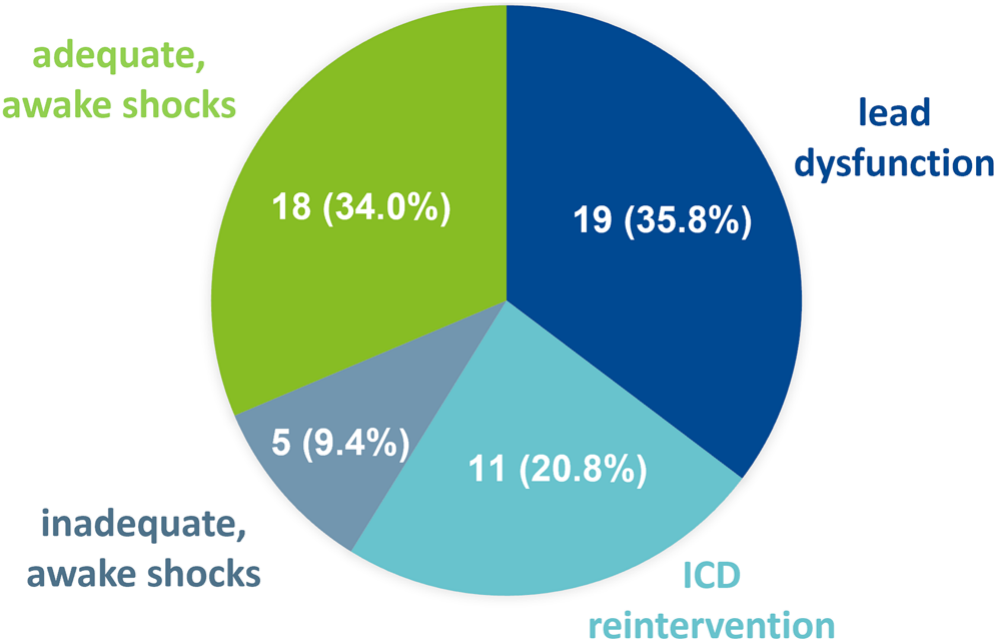
ICD-related adverse events during FU. 53 ICD-related AEs occurred during follow-up. Lead-dysfunction occurred in 19 patients and unplanned ICD-reintervention in 11 patients. Furthermore, in 18 patients, appropriate shocks without the loss of consciousness occurred, whereas inappropriate shocks occurred in 5 patients.

In contrast to the evident benefit of ICD implantation in symptomatic HF patients with severe LV dysfunction by preventing SCD, the prognostic effect of ICD therapy in LVAD recipients remains controversial (12). Therefore, we retrospectively analyzed 162 consecutive end-stage HF patients receiving LVAD implantation at our institution, who were categorized according to the presence or absence of ICDs prior to LVAD implantation (Figure 1). During a median FU of 14 (3.0-36.5) months ICD therapy was not associated with a survival benefit or reduced morbidity after LVAD implantation (Figure 2). In contrast, 53 ICD-related AEs occurred during FU, including 19 patients with lead-dysfunction and 11 patients who had to undergo unplanned ICD-reintervention after LVAD implantation. Furthermore, in 18 patients appropriate “awake shocks” occurred, whereas inappropriate shocks occurred in 5 patients (Figure 3).

### Study population and in-hospital outcome

Apart from comparable patient demographics and most outcome-relevant comorbidities, we found several essential differences regarding patient characteristics between both groups due to the non-randomized retrospective study-design. Thereby, despite similar echo-cardiographic parameters of LV and RV dysfunction, Control-group patients without an ICD prior to LVAD implantation, showed an increased rate of previous short-term device support and hemodialysis. Furthermore, an increased number of INTERMACS profiles ≤2 patients reflects a higher prevalence of acute cardiogenic shock within the Control-group. Thus, perioperative RHF occurred more frequently within the Control-group underlining an increased operative risk. Of note, 30-day mortality was numerically increased within the Control-group (20.6% in the Control-group vs. 12.8% in the ICD-group; *p* = 0.18). Nevertheless, multivariable adjusted Cox regression analysis could exclude independent impact of the differing baseline characteristics on survival [Table 4].

**Table 4:** Multivariable adjusted Cox regression analysis.

Prediction of survival:			
	HR	95% CI	p-value
ICD vs. Control	0.884	0.321–2.435	0.811
Ischemic Cardiomyopathy	0.985	0.485–2.003	0.967
Diabetes mellitus	0.953	0.487–1.865	0.888
Prev. Hemodialysis	0.399	0072–2.221	0.294
Serum Creatinin level	1.464	0.847–2.529	0.172
Serum GOT level	1.001	0.994–1.008	0.736
Serum GPT level	0.998	0.991–1.005	0.658
LVEDD	0.980	0.948–1.013	0.234
NYHA class IV	1.335	0.622–2.868	0.459
Previous ECMO/Impella	1.071	0307–3.737	0.914
INTERMACS class ≤ 2	0.986	0.406–2.398	0.976

HR, hazard ratio; CI, confidence interval.

In contrast to a higher prevalence of acute deterioration within the Control-group, one might speculate, that ICD-group patients suffered from a prolonged history of chronic HF. Consequentially, an increased LVEDD within the ICD-group prior to LVAD implantation might point out excessive adverse LV remodeling in the ICD-group in comparison to the Control-group. Furthermore, we found a higher rate of reverse remodeling resulting in LVAD explantation within the Control-group.

### Two-year outcome and ICD-associated AEs during FU

VAs occur frequently in patients with HF and severe LV dysfunction, leading to an impaired patient outcome (13, 14). Apart from ischemia-induced arrhythmogenic substrates, myocardial fibrosis due to non-ischemic dilated cardiomyopathy increases the risk of VAs as well (15). Interestingly, excessive LV dilatation accompanying advanced adverse LV remodeling, is associated with an increased rate of VAs and SCD in patients with end-stage HF (16). In addition, LVAD recipients exhibit an increased risk of early and late VAs after LVAD implantation due to proarrhythmic effects of inotropic agents, electrolyte imbalance and mechanical interaction between myocardium and the pump (e.g., suction effect) (9, 10, 17). Furthermore, previous VAs and a longer history of HF prior to LVAD implantation, as well as the type of underlying cardiomyopathy represent strong predictors of recurrent VAs after LVAD implantation (13, 18).

Contradictory data regarding the prognostic impact of VAs and ICD therapy in LVAD recipients has been published. Whereas a survival benefit of ICD implantation in pulsatile flow LVAD recipients has been described (19, 20), the prognostic effect of VAs and ICD therapy in cf LVAD recipients remains questionable. Mainly due to the ongoing cardiac output via the continuous flow of the pump, the risk of SCD due to sVT or VF seems to be negligible (21). Nevertheless, ongoing VAs might increase the risk of RHF, which is associated with rehospitalization and an impaired survival in this patient cohort (17).

Although approximately one third of ICD-group patients had recurrent VAs within the first two years after LVAD implantation, we found no overall survival benefit during FU in comparison to the Control-group. Of note, ICD-group patients were potentially at a higher risk of developing VAs after LVAD implantation due to an increased number of previous VAs and a longer history of HF with a potentially increased extend of adverse LV remodeling. Nevertheless, the prevalence of cardiogenic shock and the rate of perioperative RHF was higher within the Control-group.

Apart from the potential benefit of ICD therapy, namely preventing SCD, it is accompanied with several AEs and complications (22, 23). During the first 2 years after LVAD implantation 53 ICD-related AEs occurred in 40 patients (42.5%). In line with Thomas et al. (22), a significant number of ICD-group patients (20.2%) developed lead-dysfunction, leading to an increased risk of VA-under- or -oversensing and inappropriate shocks (24). Furthermore, 12% of patients had to undergo unplanned ICD-reintervention. Thereof, RV-lead revision due to immediate RV-lead dysfunction after LVAD-implantation and re-operation due to pocket hematoma and infection after elective generator exchange were most prevalent in our study-cohort. Due to the mandatory anticoagulation in LVAD recipients, perioperative bleeding and pocket hematoma after generator exchange occur frequently (25, 26), and are associated with an increased risk of bacteriemia and infection (27). As a beneficial prognostic effect of ICDs in cf LVAD recipients is yet to be proven, routine generator replacement remains debatable (28) and the potential risk of hematoma and infection should be taken into consideration during shared decision making to avoid unnecessary complications. Of note, 43.6% (41/94) of ICD-group patients underwent CRT-D implantation prior to LVAD-implantation. As there are conflicting reports regarding the benefits of biventricular pacing in LVAD recipients (1), LV lead settings were maintained after LVAD implantation, unless LV lead dysfunction occurred, and reprogramming was mandatory. Given the fact, that biventricular stimulation potentially results in a more rapid battery depletion and an increased necessity of generator exchanges, deactivation of LV stimulation can be discussed.

Approximately one third of ICD-group LVAD recipients (27,7%) experienced significant VAs, whereof 18 patients had appropriate shocks without the loss of consciousness. Additionally inappropriate “awake shocks” occurred in 5 patients. In contrast to HF patients without LVAD support, ICD shocks might not be associated with an increased risk of mortality (29). Nevertheless, even without an impaired survival, ICD shocks, particularly without a loss of consciousness, are potentially associated with posttraumatic stress and an impaired quality of life of the affected patients (30). Therefore, particularly in patients with previous refractory VAs, who underwent sec. prevention ICD-implantation, electrophysiological studies and catheter-based VT ablation prior to, or after LVAD implantation, as well as surgical VT ablation during LVAD implantation, might become a valuable therapeutic option.

Due to the heterogeneity of different devices implanted (e.g., single-chamber, dual-chamber and CRT-D), as well as implantation strategies (primary vs. secondary prevention), different device settings have been present within the ICD-group prior to LVAD-implantation. To avoid ICD-shocks without the loss of consciousness, conservative ICD-programming strategies, that minimize ICD shocks, were used in the ICD-group after LVAD-implantation. For example, maximally extended detection intervals prior to ICD-shock and optimizing the use of ATP for VTs was preferred. Furthermore, in 9 patients deactivation of ICD-therapy was performed due to the requests of the patients, after they experienced adequate or inadequate shocks without the loss of consciousness. Therefore, a case-by-case discussion, including the type of the device, device strategy (primary vs. secondary prevention) and patients' history (e.g., s/p refractory VAs etc.) is mandatory, to determine post-LVAD ICD-settings to further improve patient outcome.

### Study limitations

The authors are aware of the non-randomized retrospective study-design limited to a single center. Due to the categorization of patients according to the presence or absence of ICD prior to LVAD implantation, essential differences regarding the perioperative risk (e.g., INTERMACS profiles), the timeframe of HF history leading to LVAD implantation, and the occurrence of previous VAs are present between groups, potentially confounding survival analysis. The risk of VAs after LVAD implantation and their clinical implications leading to a potential prognostic benefit in ICD-group patients, might be affected. Furthermore, due to the retrospective study design, analysis of the original ventricular intracardiac electrograms (EGMs) of the detected late VAs and characterization of VT-morphology and cycle length was not possible.

To minimize further confounding, pulsatile flow and durable RVAD patients were excluded. Furthermore, despite the lack of a uniform “programming-protocol”, which is of highest importance to standardize device-programming in LVAD-recipients, there were no major changes regarding ICD-programming and follow-up schedule. Of note, we achieved a complete data collection and FU.

## Conclusion

ICD therapy in LVAD recipients was not associated with a survival benefit or reduced morbidity after LVAD implantation. Despite the lack of a randomized controlled trial conservative ICD-programming seems to be justified to avoid ICD-related complications and „awake shocks” after LVAD implantation. Furthermore, an increased risk of hematoma and infection after planned or unplanned ICD-reintervention should be taken into consideration during shared decision making to avoid unnecessary complications in LVAD recipients.

## Data Availability

The raw data supporting the conclusions of this article will be made available by the authors, without undue reservation.
