# Supplementary material for: Prognostic impact of implantable cardioverter defibrillators and associated adverse events in patients with continuous flow left ventricular assist devices
Source: Front Cardiovasc Med. 2023 Jun 2;10:1158248. doi: 10.3389/fcvm.2023.1158248 (PMC10272823; doi:10.3389/fcvm.2023.1158248)
Supplement: Supplementary file 1 [file Table1.docx]

**Supplementary material**

**Supplementary Table S1: Subgroup patient characteristics and perioperative outcome**

| **Variables** | **Primary prev. ICD**  **(n= 57)** | **Secondary prev. ICD**  **(n= 37)** | **p-Value** |
| --- | --- | --- | --- |
| Age (years), median (IQR) | 56 (46-61) | 59 (52-66) | 0.023 |
| Male, n (%) | 47 (82.5) | 32 (86.5) | 0.60 |
| BMI, kg/m^2^, median (IQR) | 28 (24-31) | 26 (23-29) | 0.38 |
| Ischemic Cardiomyopathy, n (%) | 22 (38.6) | 14 (37.8) | 0.94 |
| Arterial hypertension, n (%) | 26 (45.6) | 13 (35.1) | 0.31 |
| Diabetes mellitus, n (%) | 26 (45.6) | 7 (18.9) | 0.007 |
| COPD > GOLD II, n (%) | 11 (19.3) | 3 (8.1) | 0.14 |
| Atrial fibrillation, n (%) | 29 (50.9) | 21 (56.8) | 0.58 |
| Previous stroke, n (%) | 8 (14.0) | 7 (18.9) | 0.53 |
| Previous hemodialysis, n (%) | 6 (10.5) | 2 (5.4) | 0.39 |
| Serum Creatinin level (mg/dl), median (IQR) | 1.7 (1.2-2.2) | 1.9 (1.4-2.4) | 0.20 |
| Serum NT-proBNP level (pg/l), median (IQR) | 8297 (3268-14006) | 6376 (3911-11214) | 0.28 |
| Serum GOT level (U/l), median (IQR) | 29 (19-53) | 28 (18-48) | 0.64 |
| Serum GPT level (U/l), median (IQR) | 30 (14-61) | 24 (13-39) | 0.50 |
| LVEF (%), mean ± SD | 20.7 ± 6.5 | 20.2 ± 6.0 | 0.85 |
| LVEDD (mm), mean ± SD | 73.1 ± 11.1 | 72.5 ± 10.9 | 0.36 |
| TAPSE (mm), mean ± SD | 15.6 ± 4.3 | 13.6 ± 4.7 | 0.20 |
| Destination therapy, n (%) | 18 (31.6) | 14 (37.8) | 0.53 |
| NYHA class IV, n (%) | 33 (59.9) | 23 (62.2) | 0.68 |
| Previous ECMO/Impella, n (%) | 8 (14.0) | 5 (13.5) | 0.94 |
| INTERMACS class ≤ 2, n (%) | 21 (36.8) | 10 (27.0) | 0.38 |
| Previous sternotomy, n (%) | 16 (28.1) | 12 (32.4) | 0.65 |
| Full-sternotomy, n (%) | 39 (68.4) | 29 (78.4) | 0.29 |
| Implantation of HVAD device, n (%) | 47 (82.5) | 34 (91.9) | 0.20 |
| Implantation of tRVAD, n (%) | 10 (17.5) | 6 (16.2) | 0.87 |
| Concomitant procedures, n (%) | 28 (49.1) | 22 (59.5) | 0.33 |
| Duration of surgery (min), median (IQR) | 283 (234-356) | 300 (255-395) | 0.29 |
| Cardiopulmonary bypass time (min), median (IQR) | 135 (109-167) | 135 (114-176) | 0.51 |
| Postoperative ventilation time (h), median (IQR) | 7 (5-11) | 9 (7-13) | 0.61 |

BMI: body mass index; COPD: chronic obstructive pulmonary disease; ECMO: extracorporeal membrane oxygenation; GOLD: Global Initiative for Chronic Obstructive Lung Disease; INTERMACS: Interagency Registry for Mechanically Assisted Circulatory Support; LVEDD: left ventricular end-diastolic diameter; LVEF: left ventricular ejection fraction; NYHA: New York Heart Association; NT-pro-BNP: N-terminal pro-B natriuretic peptide; GOT: glutamic oxaloacetic transaminase; GPT: glutamic pyruvic transaminase; TAPSE: tricuspid annular plane systolic excursion; tRVAD: temporary right ventricular assist device
